# Projected impacts of climate change on habitat availability for an endangered parakeet

**DOI:** 10.1371/journal.pone.0191773

**Published:** 2018-01-24

**Authors:** Claudia Hermes, Klaus Keller, Robert E. Nicholas, Gernot Segelbacher, H. Martin Schaefer

**Affiliations:** 1 Chair of Wildlife Ecology and Management, Faculty of Environment and Natural Resources, University of Freiburg, Freiburg, Germany; 2 Earth and Environmental Systems Institute, Pennsylvania State University, University Park, Pennsylvania, United States of America; 3 Department of Geosciences, Pennsylvania State University, University Park, Pennsylvania, United States of America; 4 Department of Engineering and Public Policy, Carnegie Mellon University, Pittsburgh, Pennsylvania, United States of America; 5 Fundación Jocotoco, Quito, Ecuador; University of New England, AUSTRALIA

## Abstract

In tropical montane cloud forests, climate change can cause upslope shifts in the distribution ranges of species, leading to reductions in distributional range. Endemic species with small ranges are particularly vulnerable to such decreases in range size, as the population size may be reduced significantly. To ensure the survival of cloud forest species in the long term, it is crucial to quantify potential future shifts in their distribution ranges and the related changes in habitat availability in order to assure the long-term effectiveness of conservation measures. In this study, we assessed the influence of climate change on the availability of forested habitat for the endemic El Oro parakeet. We investigated the future range shift by modelling the climatic niche of the El Oro parakeets and projecting it to four different climate change scenarios. Depending on the intensity of climate change, the El Oro parakeets shift their range between 500 and 1700 m uphill by the year 2100. On average, the shift is accompanied by a reduction in range size to 15% and a reduction in forested habitat to only 10% of the original extent. Additionally, the connectivity between populations in different areas is decreasing in higher altitudes. To prevent a population decline due to habitat loss following an upslope range shift, it will be necessary to restore habitat across a large elevational span in order to allow for movement of El Oro parakeets into higher altitudes.

## Introduction

Tropical montane cloud forests harbor some of the highest concentrations of biodiversity on Earth [1–3]. Their extreme altitudinal zonation of microhabitats combined with narrow species ranges make them centers of endemism [2, 3]. Tropical montane cloud forests depend on frequent immersion in the cloudbank, creating a cool, moist environment with low direct sunlight and low evapotranspiration [4]. Anthropogenic climate change has the potential to result in an uphill shift of the cloudbank, thus leaving lower elevation zones in hotter and drier conditions [5, 6]. Consequently, cloud forest animal and plant species depending on a humid, cool environment may experience severe climatic distress caused by warming temperatures.

Tropical species typically have narrow temperature niches and low tolerance to temperature changes [7–10]. To avoid the negative impacts of rising temperatures, species may evade into cooler surroundings. Previous work has demonstrated behavioral responses of tropical species to global warming, like poleward or upslope shifts in animal and plant communities as a consequence of increasing temperatures in their original ranges [11–13]. In tropical montane cloud forests, species may shift within their specific temperature niche uphill at a speed of up to 70 m per decade [14–18], while the speed of the shift lags behind temperature increase [19]. In comparison to species in temperate zones, which have been observed to shift their ranges at a rate of 4 to 30 m per decade [20–22], tropical cloud forest species thus seem to be much more strongly affected by warming temperatures. Since tropical mountains are centers of endemism and species often have only narrow altitudinal ranges, upslope range shifts can result in a complete exchange of the ecosystem in lower zones, reductions in the size of the distribution range and extinction of species occurring near mountain tops [4]. In the case where an upslope range shift is impossible and no other suitable habitat is available, tropical cloud forest ecosystems may be at risk of severe losses in biodiversity.

The intensity of an upslope range shift is not only determined by climatic variables, but also by topographical characteristics of the area. In the case of environmental barriers such as mountain tops or fragmented habitat, a range shift might not be possible as suitable habitat is no longer available. In this case, a species has to respond to warming temperatures within the same habitat. In order to prevent local extinction due to heat stress, a species will need to adapt physiologically to increasingly warmer temperatures and expand its temperature niche. A species’ potential to adapt genetically or through phenotypic plasticity to environmental changes is largely determined by its genetic diversity [23–25]. The genetic diversity of a species, in turn, depends strongly on the population size and the level of gene flow between sub-populations in different areas [26, 27]. Therefore, sufficient habitat and connectivity among sub-populations are crucial to facilitate migration and gene flow and thereby ensure a high level of genetic diversity. As a result, conservation efforts that aim to preserve habitat and restore connectivity are required to maintain the potential of cloud forest species to adapt to warming temperatures.

The cloud forests in the global biodiversity hotspot of Tumbes-Chocó-Magdalena on the western slopes of the Andes are the habitat of more than 400 endemic animal species [2]. Ongoing deforestation strongly threatens these species with climate change being an additional threat, leading to further habitat loss and reduction of connectivity [28, 29]. Previous studies have broken important new ground by modelling climate change effects on species richness and distribution in the hotspot [30, 31]. However, thus far, we lack detailed information about the future temperature-induced upslope shift at the species level and at the southern edge of the hotspot. This information is essential because high-resolution data on range shifts are necessary not only to quantify a species’ vulnerability to warming temperatures [32], but also to examine the availability and configuration of suitable habitat in the projected range. These data can help to design conservation measures that aim at ensuring habitat availability and connectivity for cloud forest species in view of ongoing climate change.

Here we analyze past and project potential future temperature-induced upslope shifts in the Tumbes-Chocó-Magdalena hotspot and the consequences that such shifts might have on the availability of habitat. To assess the generality of our results, we investigate the relationship between altitudinal shift and forest cover at four different sites in southwestern Ecuador. We use the El Oro parakeet (*Pyrrhura orcesi*), which is endemic to the cloud forests in a narrow range in the Tumbes-Chocó-Magdalena hotspot and heavily threatened by forest loss, as a model species for this study. There is evidence that the species has shifted its range uphill in the decades since its discovery in the 1980s [33]. As the El Oro parakeets are susceptible to forest fragmentation [34], the survival of the species hinges on the availability of forest habitat within its range. The El Oro parakeet is a mobile species, but presumably not very temperature-sensitive. The range of the El Oro parakeet overlaps with the ranges of more than 350 bird species, of which 15 are endangered and 34 locally endemic [35]. We assume that the results gained for the El Oro parakeet and the conservation implications derived from these results are also applicable and of high relevance for many other cloud forest species, including such species which are less mobile, like amphibians [5, 12] or dispersal-limited bird species. Therefore, we consider it an appropriate umbrella species for assessing the upslope shift in the southern part of the Tumbes-Chocó-Magdalena hotspot.

We quantify the upslope range shift by assessing the temperature niche of the El Oro parakeet and projecting it for four different climate change scenarios as described by the Intergovernmental Panel on Climate Change’s Fifth Assessment Report [36]. Then, we transfer the projected ranges to a map of forest fragments in the El Oro parakeet’s distribution range. We hypothesize that habitat availability differs between climate change scenarios, depending on the amount of the range shift. We expect the differences in habitat availability to be predictable, with an inverse relationship between habitat availability and the intensity of climate change.

## Material and methods

### Study area and study species

**Fig 1:**
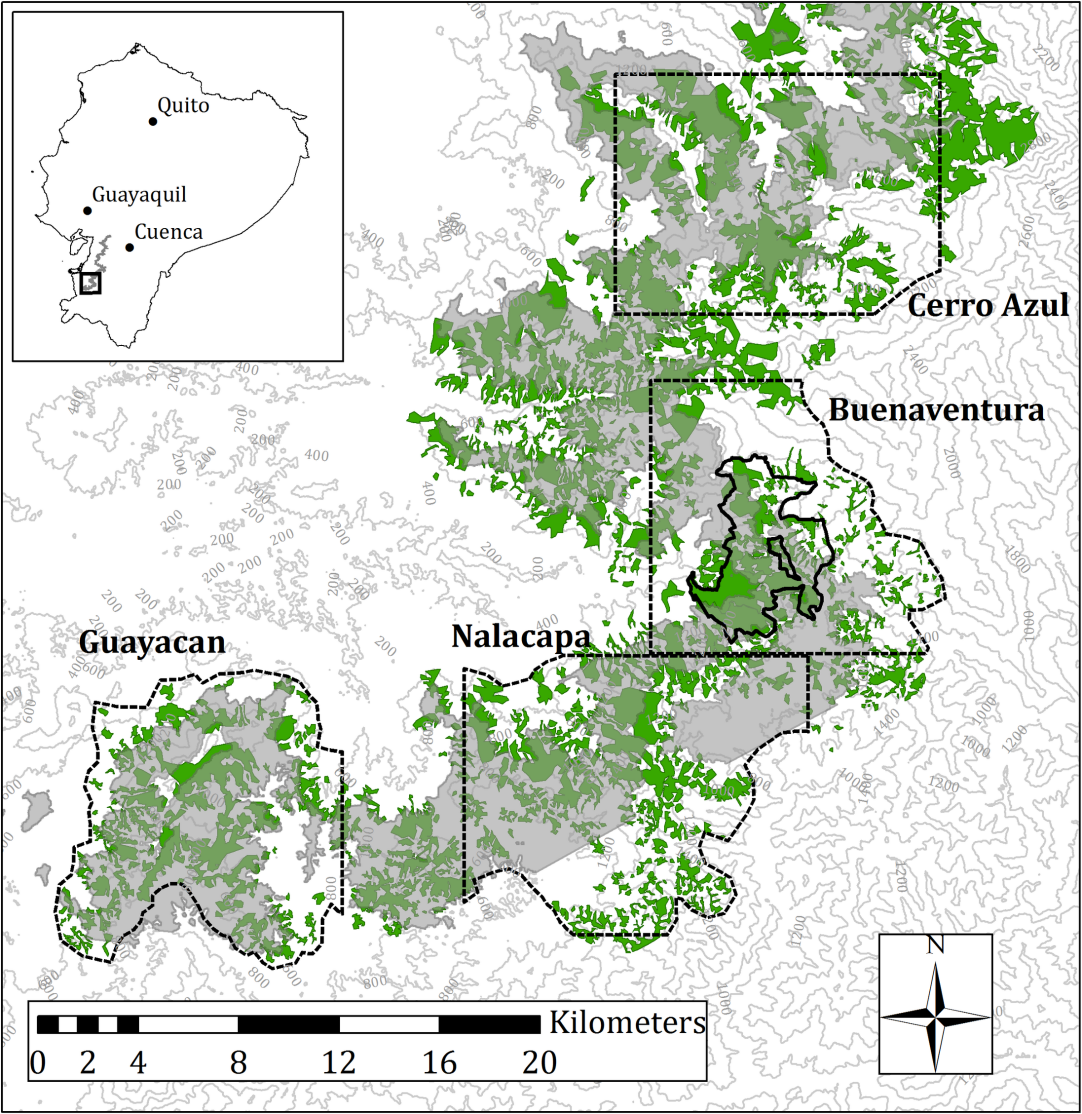
Overview of the southern part of the original distribution range of the El Oro parakeet in southwestern Ecuador. The extent of the range was inferred from the altitudinal band between 600–1100 m (grey), where the El Oro parakeet was discovered. Forest fragments in the area are depicted in green. The four study areas of Cerro Azul, Buenaventura, Nalacapa and Guayacan are indicated by the dashed outlines, with the Buenaventura reserve (solid black outline) being the only protected site within the area.

The El Oro parakeets are endemic to a small range (~ 750 km^2^) in the southernmost cloud forests of the Tumbes-Chocó-Magdalena hotspot (Fig 1). This hotspot is located on the western slopes of the Andes and stretches from Panama through Colombia and Ecuador to northern Peru. Over 2500 plant species and 400 vertebrate species are endemic to the hotspot [2]. Importantly however, the Tumbes-Chocó-Magdalena hotspot is facing an exceptionally high projected rate of habitat loss. Model simulations suggest that by the end of the 21^st^ century, the hotspot will have lost around 90% of its original area due to global warming and land-use change [29].

The distribution range of the El Oro parakeets is affected by intense deforestation. Up to 95% of the original forest cover in western Ecuador has already been logged and converted into cattle pasture [37]. The only protected site within the parakeets’ distribution range is the Buenaventura reserve near Piñas (S 3,655°, W 79,744°). This reserve covers an area of 2575 ha of secondary forests and abandoned cattle pastures in altitudes between 400 and 1450 m. Outside the reserve, remaining forest fragments are mostly small (< 100 ha) and separated by large areas of open habitat (Fig 1). As a result of the intense deforestation throughout its range, the population of the El Oro parakeet is declining rapidly. The population size is currently estimated at less than 1000 mature individuals [38]. Being frugivorous birds breeding in tree cavities, the El Oro parakeets rely heavily on a dense forest cover [34]. However, the species is able to cope with a certain degree of forest fragmentation and can cross gaps of up to 600 m between forests [34, 39]. The El Oro parakeet was discovered in the 1980s, when it occurred at an altitude of 600–1100 m, with one record from 1931 at 300 m [40]. Since then, the El Oro parakeets have continuously shifted their distribution range uphill: In 2003, they occurred mainly at 800–1300 m [34], while in 2016 they could occasionally be observed in altitudes as high as 1600 m [33]. It has been hypothesized that the upslope shift could be attributed to increasingly warmer and drier conditions in the lower elevation zones [33].

### Climate data

We obtained detailed climate data for the area of the Buenaventura reserve (box coordinates: 3,616°S–3,675°S; 79,738°W–79,782°W) from KNMI Climate Explorer [41]. For our study, we focused on the anomalies of annual mean temperature and annual precipitation sum for the years 2000–2100 (reference period 1986–2005). We obtained historical climate data (years 2000–2015) from the ERA-interim reanalysis [42]. The ERA-interim reanalysis reconstructs a high-resolution model of the state of the atmosphere from 1979 to the present. Future climate (years 2016–2100) was expressed by a subset of CMIP5 models used in the Atlas of Global and Regional Climate Projections (hereafter Atlas subset) of the IPCC’s Fifth Assessment Report [36]. We used the output of multiple models to account for key uncertainties due to factors such as different model structures, model parameters, and model initial conditions (for a discussion, see for example [43]). Future climate forcings were sampled using results for four Representative Concentration Pathways: RCP2.6, RCP4.5, RCP6.0, and RCP8.5. The RCPs describe different trajectories for greenhouse gas emissions, leading to specific values of radiative forcing by the year 2100 (2.6, 4.5, 6.0, and 8.5 W/m^2^). While RCP2.6 represents a scenario of strong greenhouse gas mitigation in the future with a likely increase of global mean temperature by ~1.5°C above pre-industrial level by the year 2100, RCP8.5 represents a scenario of drastically increasing climate forcing, with a projected temperature increase of ~4.9°C by 2100 (sometimes referred to as “business as usual”). RCP4.5 (temperature increase of ~2.4°C by 2100) and RCP6.0 (temperature increase of ~3.0°C by 2100) describe intermediate scenarios [36]. The models used for each RCP scenario are listed in the Supporting Information (S1 Table; see also [36]).

### Modelling the climatic niche of the El Oro parakeet and projecting the range shift

El Oro parakeets were observed by teams of 1–3 observers on 50 observation sites in the rainy season (December to July) of the years 2002, 2011 and 2012 in the Buenaventura reserve in elevations between 800 and 1300 m using consistent methods according to pre-established protocols (see [44]). Surveys were always done on multiple days. Each day, observations started at 06:30 and ended at 17:00. The 50 observation sites remained constant across the years. Observation sites were chosen based on openness and visibility (hilltops, look-out points, etc.). All observations of parakeets were recorded in an unlimited radius, with point counts at fixed time intervals. Fly-bys were counted, and the altitude and direction of flight noted and compared among observers. If a same-sized group was recorded by another observer in the direction of flight, it was not counted twice to avoid over-estimating the population. As our study was purely observatory and did not involve any handling or sampling of birds, no specific permission was required.

**Fig 2:**
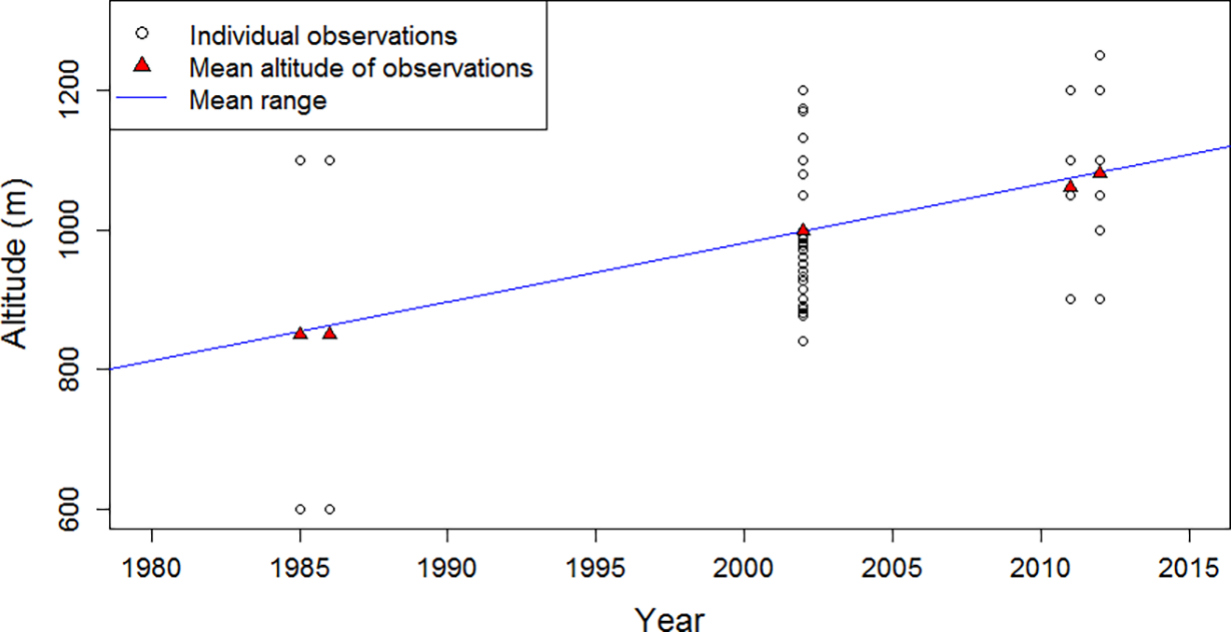
Altitudes of El Oro parakeets. The birds were observed in the years 2002 (998 ± 36 m), 2011 (1061 ± 63 m), and 2012 (1081 ± 23 m). For the years 1985 and 1986, literature values of the distribution range are given (600–1100 m). The El Oro parakeets shifted their range uphill at a rate of 8.5 m per year (depicted by the blue line).

In 2002, the birds occurred at an altitude of 998 ± 36 m (mean ± standard deviation; n = 353 observations), while in 2011 (n = 70 observations) and 2012 (n = 1063 observations) we found them at 1061 ± 63 m and 1081 ± 23 m, respectively (Fig 2). The mean altitudes for 2002, 2011, and 2012 were approximately equal to the average of the extreme values (2002: 1020 m; 2011: 1050 m; 2012: 1075 m). We assessed whether climate variability influenced the altitude of records of the El Oro parakeets over time using simple statistical models. To quantify the shift in their altitudinal range, we fitted a simple linear model of the altitude as a function of time (altitude = altitude_0_ + α * Δ_Year_), using the means of the observed altitudes for the years 2002, 2011 and 2012. Additionally, we included the means of the altitudinal ranges for the years 1985 and 1986 (850 m; [40]). We neglected the effects of uncertainties in altitude and of different sample size between the years. A detailed analysis of the uncertainties of the model is beyond the scope of this paper. For the linear regression, we assumed that errors are independent, as well as normally and identically distributed. The least square estimate of this simple model resulted in an uphill movement of the distribution range of the El Oro parakeets of 8.5 m per year (*R*^*2*^ = 0.997; *p* < 0.001).

To assess the influence of climate parameters on the upslope shift of the El Oro parakeets, we first carried out a linear regression to evaluate the variability in temperature and precipitation anomalies over the study period (temp = temp_0_ + α * Δ_Year_ and precip = precip_0_ + α * Δ_Year_; null hypothesis: no trend in climate parameters over time). We found a positive linear trend in temperature over the years (*R*^2^ = 0.730; *p* < 0.1), but no trend in precipitation (*R*^*2*^ = 0.484; *p* > 0.1). Additionally, we assessed the relationships between temperature and precipitation anomalies and the mean altitude of El Oro parakeets for the five years 1985, 1986, 2002, 2011, and 2012 (altitude = altitude_0_ + α * Δ_temp_ and altitude = altitude_0_ + α * Δ_precip_; null hypothesis: no altitudinal trend in climate parameters). Likewise, altitude was positively correlated with temperature (*R*^*2*^ = 0.742; *p* < 0.1), but not with precipitation (*R*^*2*^ = 0.502; *p* > 0.1). These correlations are consistent with the hypothesis that temperature anomalies are the important drivers of altitude of El Oro parakeets. We hence approximated the situation with a simple model in which temperature alone influences the upslope shift, neglecting uncertainties in altitudes and temperature. All further analyses thus were conducted for temperature anomalies only.

In the linear regression of temperature anomalies and mean altitudes in a given year, we obtained an estimate for the temperature niche of the parakeets. To predict the geographic extent of the El Oro parakeets’ distribution range under different climate change scenarios, we projected the temperature niche to the Atlas subset models for each of the four RCPs.

### Projected future habitat availability and connectivity

We quantified changes in the geographical range and habitat availability for the El Oro parakeet over an area covering about one-third of the species’ distribution range. To this end, we projected the original range (600–1100 m) and the expected ranges for the years 2050 and 2100 to a map of forest fragments in ArcMap 10.2. The map was created using satellite images (RapidEye, Blackbridge, Germany) with a resolution of 5 m from the years 2010 and 2013 as a template; forested habitats ≥ 0.1 ha were mapped manually in ArcMap 10.2. Prior to the analysis, we identified four study areas Cerro Azul (CA), Buenaventura (BV), Nalacapa (NA) and Guayacan (GY, Fig 1), where parakeets are known to occur. The exact extent of the study areas (each ~ 100 km^2^) depended on the availability of forest maps. Within each of the four areas, we measured the size of the original altitudinal range and the projected ranges for 2050 and 2100 under the four RCP scenarios. We assumed the range of the El Oro parakeet to span 500 m in altitude, implying a parallel shift of the lower and upper range boundaries. Additionally, we measured the extent of forested habitat within the original and projected ranges. To assess differences in the connectivity between the forest fragments, we measured the shortest distances between forest fragments within the original and projected ranges using the “Near” tool of ArcMap 10.2. We based this analysis on the assumption that no changes in the extent and distribution of forest cover would occur until the year 2100. This assumption relies on forest cover not being limited by climate, but by anthropogenic land use. We acknowledge that this assumption is simplistic and most likely not plausible considering the high deforestation rates in the area; however, it yields information about the order of magnitude in the changes in habitat availability that is to be expected under the different climate change scenarios.

## Results

### Modelling the climatic niche of the El Oro parakeet

**Fig 3:**
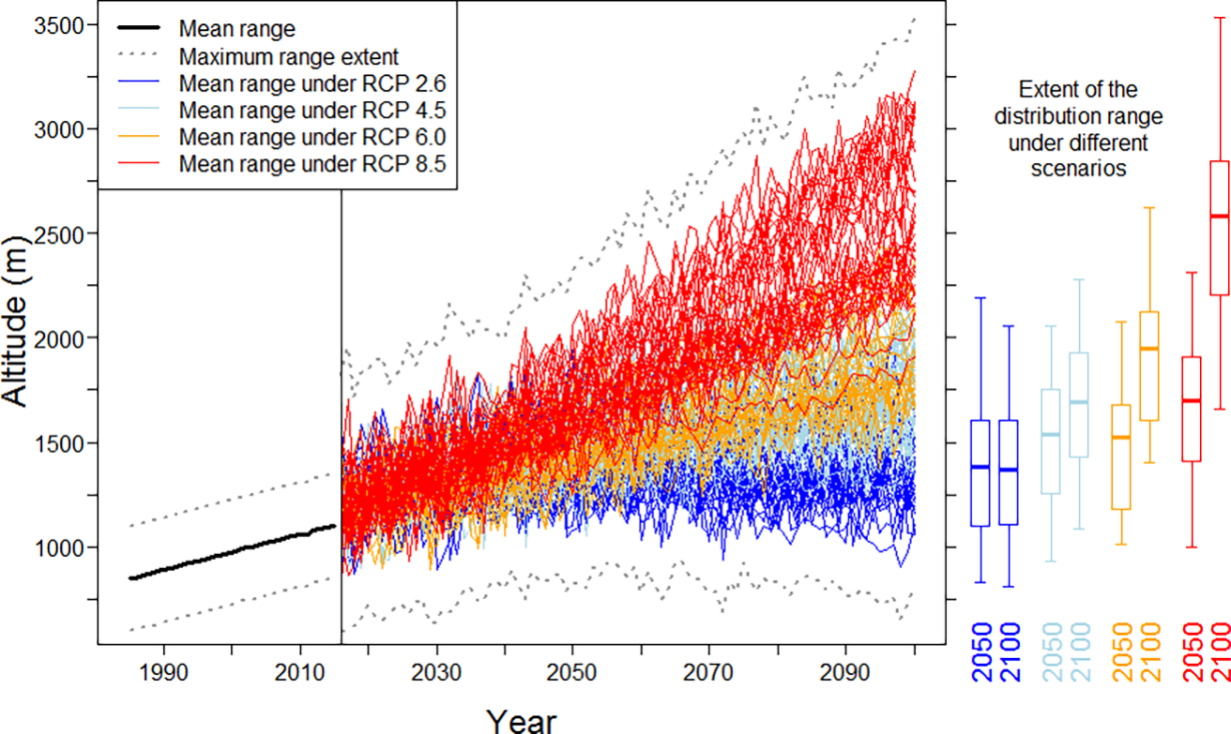
Projected upslope shift of the El Oro parakeets’ distribution range between the years 2000 and 2100. The bolt black line indicates the altitudes of parakeets between 1985 and 2015. Each colored line represents the mean altitude of parakeets derived from the single CMIP5 model runs for the four scenarios RCP2.6, 4.5, 6.0, and 8.5, for the years 2016 to 2100. The maximum extent of the distribution range (mean ± 250 m) is shaded grey. Boxplots indicate the altitudinal distribution of parakeets in the years 2050 and 2100 for each of the four scenarios RCP2.6, 4.5, 6.0, and 8.5.

The simple linear regression suggests that between the years 1985–2015, El Oro parakeets shifted their range uphill by roughly 415 m per 1°C warming (Pearson’s product moment correlation: r = 0.862; *p* < 0.1; null hypothesis: no correlation between altitude and year). While in 1985 they occurred at a mean altitude of 850 m, according to our model by 2015 they already moved uphill to 1110 m (Fig 3, S1 Fig).

### Projection of the range shift

**Fig 4:**
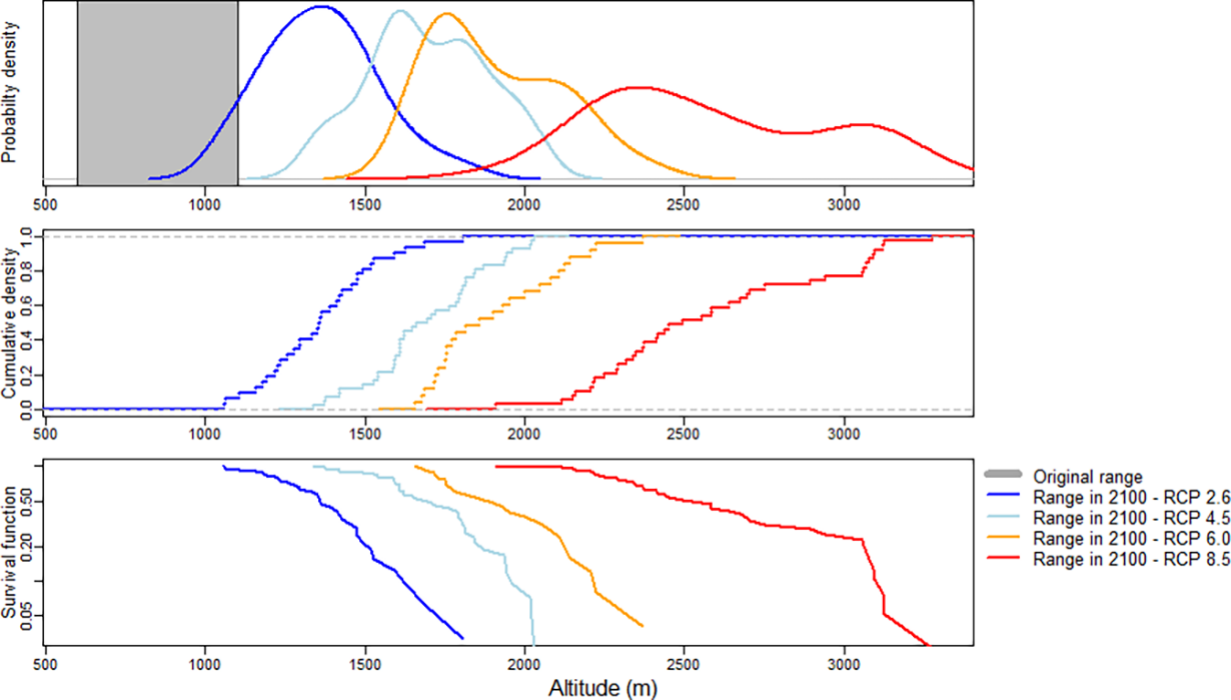
Probability density, cumulative density and survival function for the four scenarios RCP2.6, 4.5, 6.0, and 8.5 for the year 2100. The altitudinal extent of the original range (600–1100 m) is shaded grey.

All four climate change scenarios implied an upslope shift of the El Oro parakeets’ distribution range by the end of the 21^st^ century (Fig 3). For the RCP2.6 climate forcing scenario, the projected shift was smallest and stagnated in the second half of the 21^st^ century: In 2050 and 2100, the parakeets’ niche occurred in the same altitude of 1110–1610 m (mean projected range ± 250 m). Under the RCP4.5 and RCP6.0 climate forcing scenarios, the shift was more pronounced (1270–1770 m in 2050 and 1450–1950 m in 2100 under RCP4.5; 1210–1710 m in 2050 and 1660–2160 m in 2100 under RCP6.0). Even though the RCP4.5 scenario implies smaller climate forcing in the long run than RCP6.0, the upslope shift under RCP4.5 was more obvious until the year 2050. The strongest considered climate forcing scenario (RCP8.5) yielded the most drastic projected upslope shift: By 2050, the distribution range would shift to an altitude of 1430–1930 m, reaching 2330–2830 m by 2100. The probability density, cumulative density, and survival function of the modelled altitudes under the four RCPs for the year 2100 are shown in Fig 4.

### Projected future habitat availability and connectivity

**Fig 5:**
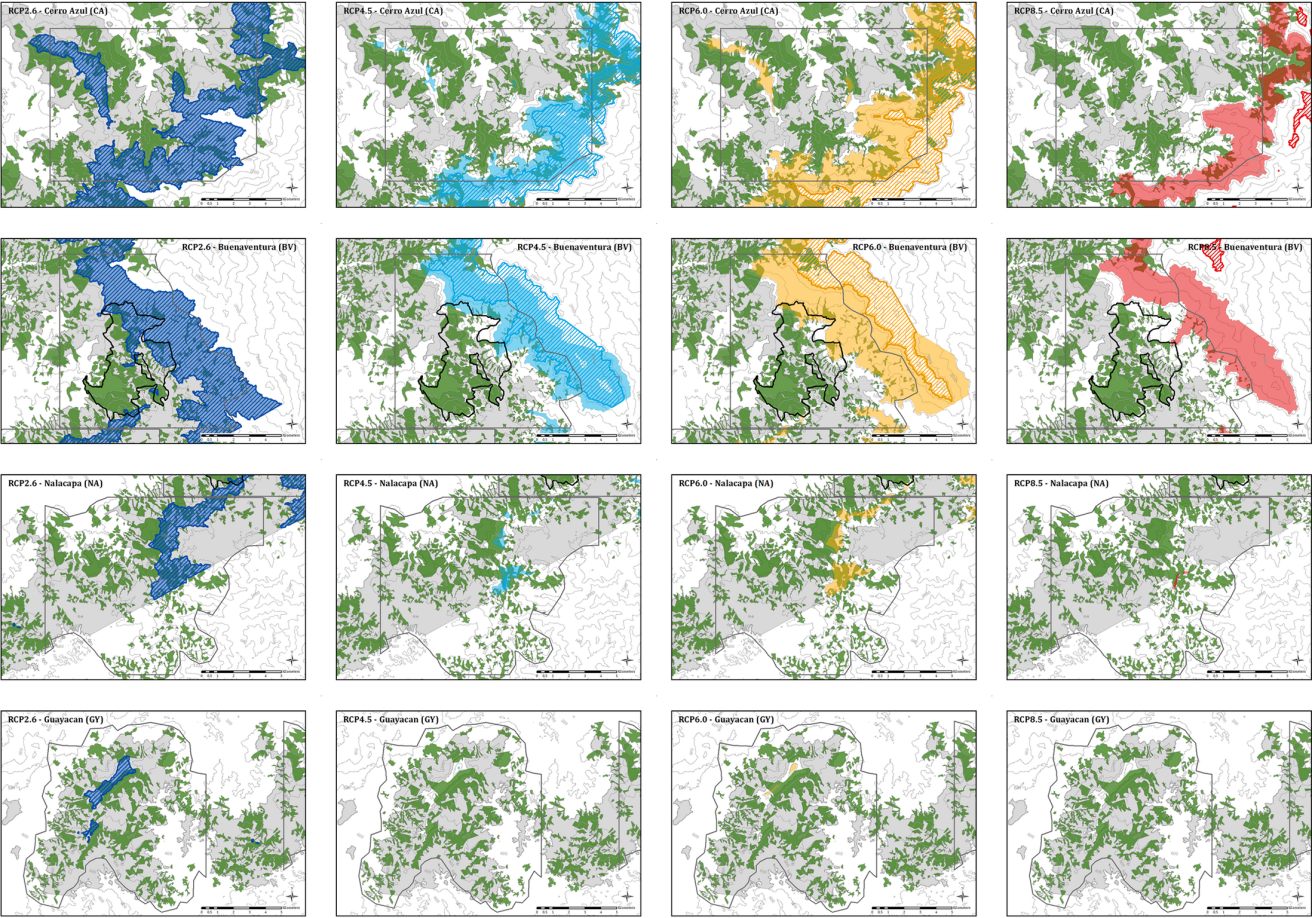
Original range and predicted ranges. The original range (light grey) and the predicted ranges for the years 2050 (intense color) and 2100 (hatched) are depicted for four climate change scenarios in the four study areas of Cerro Azul (top row), Buenaventura (second row), Nalacapa (third row), and Guayacan (bottom row). Ranges predicted for the RCP2.6 scenario are colored dark blue, for the RCP4.5 scenario light blue, for the RCP6.0 scenario orange, and for the RCP8.5 scenario red. Forests fragments are shaded green.

The size of the geographical distribution range of the El Oro parakeets is projected to decrease within all of the four scenarios by the year 2100. Under the most optimistic scenario RCP2.6, the range contraction varied between 5% in Guayacan (GY) and 98% in Buenaventura (BV) of the original range. Under RCP4.5, the projected range decreased to 20% in Cerro Azul (CA) to 31% in BV, while no habitat remained in Nalacapa (NA) and GY. Under RCP6.0, the projected range shrank to 6% in CA to 9% in BV, with no habitat remaining in NA and GY. Under RCP8.5, the geographic range completely retracted from the four study areas (Table 1, Fig 5).

**Table 1:** Habitat availability and connectivity for the original range and the projected ranges for 2050 and 2100 under four RCP scenarios.

		Original	RCP2.6		RCP4.5		RCP6.0		RCP8.5	
			2050	2100	2050	2100	2050	2100	2050	2100
Altitude [m]		600–1100	940–1440	940–1440	1030–1530	1130–1630	990–1490	1250–1750	1120–1620	1630–2130
Cerro Azul CA (12120 ha)	Altitudinal range size [ha]	5880	3930 (67%)	3930 (67%)	2250 (38%)	1190 (20%)	2830 (48%)	340 (6%)	1290 (22%)	0 (0%)
	Forested area [ha]	2520	1130 (45%)	1130 (45%)	500 (20%)	170 (7%)	710 (28%)	10 (0%)	190 (8%)	0 (0%)
	Distance between fragments [m]	120	80 (67%)	80 (67%)	130 (108%)	170 (142%)	90 (75%)	800 (667%)	130 (108%)	
Buenaventura BV (9880 ha)	Altitudinal range size [ha]	4030	3930 (98%)	3930 (98%)	2480 (62%)	1250 (31%)	2920 (72%)	380 (9%)	1370 (34%)	0 (0%)
	Forested area [ha]	1650	750 (45%)	750 (45%)	290 (18%)	150 (9%)	400 (24%)	110 (7%)	140 (8%)	0 (0%)
	Distance between fragments [m]	90	100 (111%)	100 (111%)	140 (156%)	130 (144%)	120 (133%)	10 (11%)	200 (222%)	
Nalacapa NA (11680 ha)	Altitudinal range size [ha]	5490	1130 (21%)	1130 (21%)	260 (5%)	10 (0%)	500 (9%)	0 (0%)	20 (0%)	0 (0%)
	Forested area [ha]	1570	560 (36%)	560 (36%)	150 (10%)	0 (0%)	290 (18%)	0 (0%)	10 (0%)	0 (0%)
	Distance between fragments [m]	110	60 (55%)	60 (55%)	120 (109%)		70 (64%)		60 (55%)	
Guayacan GY (10070 ha)	Altitudinal range size [ha]	5500	270 (5%)	270 (5%)	0 (0%)	0 (0%)	20 (0%)	0 (0%)	0 (0%)	0 (0%)
	Forested area [ha]	1960	130 (7%)	130 (7%)	0 (0%)	0 (0%)	10 (1%)	0 (0%)	0 (0%)	0 (0%)
	Distance between fragments [m]	90	210 (233%)	210 (233%)						

Habitat availability is given as the area of the altitudinal range and as the forested area within the respective range. Connectivity is given as the mean distances between forest fragments within the respective range. Values in parentheses indicate the percentage of the original range.

The forested area within the projected ranges decreased more drastically than the range size. While in the range projected for 2100 under RCP2.6, the forested area decreased to between 7% (GY) and 45% (CA and BV) as compared to the extent in the original range, the forested area amounted to 7% (CA) to 9% (BV) of the original extent under RCP4.5. Under RCP6.0, the area covered by forest is projected to be more drastically reduced (7% in BV, no forest in the projected ranges in CA, NA, and GY; Table 1, Fig 5).

The change in connectivity between forest fragments showed no congruent pattern. In the range projected for 2100 under RCP2.6, the distance between fragments varied between 55% (NA) and 233% (GY) as compared to the original range. Under RCP4.5 the distances increased to 142% (CA) and 144% (BV). Under RCP6.0, the distances decreased in BV (11%), but increased largely in CA (667%; Table 1, Fig 5).

## Discussion

We quantified the projected upslope shift in the El Oro parakeet’s distribution range during this century for different climate change scenarios. Depending on the intensity of climate forcing and projected climate change, the predicted range shifts between 500 and 1700 m uphill by the end of the 21^st^ century. Importantly, this shift is accompanied by a drastic loss in habitat for the species and for the ecosystem. Moreover, the shift will additionally disrupt connectivity between parakeet populations in different areas.

### Upslope shift of the distribution range

Previous studies have identified temperature increase as the primary driver for species’ range shifts [12, 45]. Consistent with this interpretation, the El Oro parakeets are moving uphill as temperatures throughout its range are warming. Between the years 1985 and 2015, the species has shifted its range uphill at an average speed of roughly 90 m per decade, corresponding to a shift of approximately 420 m per 1°C warming. El Oro parakeets have been observed to seasonally migrate between the lower and higher areas in the Buenaventura reserve, which might be caused by a low tolerance to temperature changes. While El Oro parakeets can be observed in lower altitudes in the cooler dry season (August-November), in the warmer wet season they stay in higher and thus cooler zones. It is conceivable that this migration is linked to the seasonal temperature variation, causing the El Oro parakeets to follow the temperature gradient. This interpretation is consistent with our finding that temperature is already now affecting the movement of the El Oro parakeets.

The upslope shift in the El Oro parakeets’ range is likely not induced by habitat loss and deterioration in lower elevations. Particularly in the Buenaventura reserve, forest quantity and quality in the lower zones increased largely within the last two decades [35]. Yet, the El Oro parakeets by now avoid the lower elevations, which even goes as far as to disrupt gene flow between populations on both sides of the valley [33]. Alternatively, the shift could also be caused by changes in food availability which may be temperature-sensitive, and may therefore be an indirect effect of the temperature increase. Previous studies have suggested that food availability might be partly responsible for altitudinal migrations of frugivorous birds [46]. However, in the El Oro parakeets’ range, a similar upslope shift has been observed in an ecologically distinct species, the endangered and endemic Ecuadorian tapaculo (*Scytalopus robbinsi*). The Ecuadorian tapaculo is a dispersal-limited, insectivorous bird which seems to have undergone a 250 m upslope shift in its distribution range within the last 25 years [47]. The altitudinal shift of a similar magnitude among two ecologically distinct species suggests that the shift in the range of the El Oro parakeets is indeed driven by sensitivity to the temperature increase rather than to sensitivity to food availability, and is therefore a direct effect of warming temperatures. These parallel altitudinal shifts of threatened endemics suggest that our results may hold more generally for species in the Tumbes-Chocó-Magdalena hotspot.

All four climate change scenarios imply an altitudinal range shift of the El Oro parakeets by the end of the 21^st^ century. Compared to the altitudinal range of 600–1100 m where the species was discovered [40], the birds are projected to shift their range uphill at a rate between 40 and 150 m per decade until the year 2100 following a temperature increase. This rate is roughly consistent with the range shifts that have been observed for endemic animal species on mountains in Madagascar and Indonesia [14, 15, 17]. Importantly, only under the most optimistic scenario (RCP2.6) will the range shift stagnate after the year 2050 (Fig 3). However, there are doubts whether climate change mitigation can limit the temperature change to fit the RCP2.6 scenario [48, 49]. Therefore, the range shift of the El Oro parakeets will likely exceed the projections under the RCP2.6 scenario and continue at least until the year 2100.

Projecting the range shift of the El Oro parakeets in a correlative approach based purely on warming temperatures is, of course, only an approximated forecast of future changes in its distribution range. Several Andean bird species were found to migrate uphill only at 30% of the rate predicted by temperature increase as the single driver [19]. A species’ response to climate change is likely not mediated by temperature alone; yet, biological mechanisms like physiological traits, phenotypic plasticity, local adaptations, species interactions, dispersal abilities, or habitat availability should also be taken into account when modeling future range shifts [50, 51]. The above mentioned biological parameters may be able to mitigate or enhance the rate of the temperature-driven range shift [9]. Most of these data are lacking for the El Oro parakeets; thus, we only integrated habitat availability into the model. It is possible that the actual changes in the distribution range will differ slightly from our projection. However, we accounted for a substantial (albeit likely overconfident) range of uncertainty in the intensity of the shift by including the results of 25 to 42 single CMIP5 models for each RCP scenario into the analysis (Figs 3 and 4). The projection of the upslope range shift is based on precious few observations. Importantly, the rate of the upslope shift is mainly influenced by the values for the altitudinal range in the years 1985 and 1986. These data, reported in [40], were obtained during two months of fieldwork across the entire geographical range of the El Oro parakeet, when the species was found only between 600 and 1100 m. Yet, based on a museum specimen collected at 300 m in 1939, it has been suggested that by the time of the discovery of the species, the El Oro parakeet was already undergoing an upslope shift in its distribution range [40]. Moreover, opportunistic observations of El Oro parakeets between the years 2013 and 2017 suggest that the statistical simulation produced reasonable results. The projection is, of course, an approximation, yet it provides a credible estimate of the potential magnitude of the shift that may be expected under distinct climate change scenarios.

### Implications for conservation

In the tropics, climate change poses a severe risk to native biodiversity. Especially birds [9, 17, 32, 52], but also insects, amphibians, and reptiles [5, 14, 15, 53] are susceptible to rising temperatures. Increased heat causes tropical species to shift their distribution ranges uphill, which can lead to reductions in the range size [4]. Remarkably, in our study we found that the projected shift of the El Oro parakeets’ distribution range does not necessarily lead to a decrease in the range size. Depending on the topography of the terrain, under some climate change scenarios the range size increased with the upslope shift, at least on the short term. However, within all projected ranges the area covered by forests, which represent the habitat of the El Oro parakeets, drastically decreases with increasing elevation. This suggests that the disproportionally large decrease in forest availability by the year 2100 to, on average, 10% of the original extent compared to a decrease in range area to on average 15%, is a general phenomenon in the cloud forests of the southern Tumbes-Chocó-Magdalena hotspot.

The available habitat shrinks more quickly than does range area. This has important implications for conservation: A species might actually be more threatened than assumed based on the analysis of the range size alone. Not considering the changes in habitat availability might underestimate a species’ extinction risk [54, 55]. After integrating projected changes in forest availability, the reassessment of the threat status of 800 Amazonian bird species resulted in an increase in the number of species qualifying as ‘threatened’ from 3% to 10% [56]. Accounting for changes in altitudinal ranges and forest cover combined for more than 800 endemic birds from tropical biodiversity hotspots produced a more striking result: 43% of the species were found to be more threatened than currently estimated by IUCN [55]. To obtain a precise quantification of the extinction risk of the species of the Tumbes-Chocó-Magdalena hotspot, a similar approach should also be implemented.

The upslope shift of the El Oro parakeets changes the connectivity among populations. While the original range covered a continuous altitudinal band from Guayacan to the northern part of the range (Fig 1), this linkage will become interrupted between Guayacan and Buenaventura by the year 2050. It has been shown that low-elevation zones present a dispersal barrier for El Oro parakeets [33]. The elevation of the valley between the two regions is so low that it will restrict dispersal as the range shifts uphill. Gene flow between populations of the El Oro parakeet in Guayacan and in the northern part of the range therefore might become greatly reduced, leading to a reduction in genetic diversity, which inevitably presents a risk of extinction [26, 27, 57]. By the year 2100, Guayacan and Nalacapa could become prime examples of extinction at a mountain top, not only of the El Oro parakeet, but also of other cloud forest species. Thus, the area of Guayacan and Nalacapa should be surveyed for the occurrence of endemic species, e.g. orchids or amphibians.

Apart from the reduction in connectivity due to topographic barriers, the unavailability of forested habitat might additionally contribute to disrupting linkages between populations. At higher altitudes, forest cover is greatly reduced. We did not detect a clear pattern in the change in connectivity between forest patches in the projected ranges. Although the configuration of forests is site-specific, the connectivity between forest patches is generally lower at higher elevations owing to the increasing ruggedness of the terrain. Thus, the increase in fragmentation accompanying the upslope shift of the range will likely influence gene flow, as even now populations are genetically structured by valleys [33]. The effect of valleys as a barrier to dispersal is aggravating as the upslope shift continues, leading to the formation of new topographic obstacles, which might further impede the movement of El Oro parakeets.

The future availability of forest in the range of the El Oro parakeet is difficult to project. We based the analysis of forest availability in the study areas on a map of forest fragments of the years 2010 and 2013, but did not account for future changes in forest cover. Considering the high deforestation rates throughout the Tumbes-Chocó-Magdalena hotspot within the last century [37], our assumption that forest cover remained constant until 2100 is very conservative. It is likely that forest cover changes differently in the four study areas within the next decades. In the protected Buenaventura reserve, intense reforestation programs are carried out, increasing the forested area significantly. The other three study areas are unprotected; there, forest cover is likely to be reduced in the future.

We identified areas that could potentially be colonized by the El Oro parakeet in the next decades and investigated the configuration of forests therein. The overall pattern of drastic decrease in forest cover in the areas that might be colonized by the El Oro parakeets in the near future already requires action to be taken in order to provide the species with forested habitat. Andean trees and vegetation communities have been reported to move uphill at 25–35 m per decade in the course of climate change [16, 58]. Compared to the El Oro parakeets, which likely move uphill at a rate of 40–150 m per decade, the migration rate for trees lags behind. It has been shown that secondary forests and even abandoned cattle pastures have the potential to regain characteristics of old-growth forests within a few decades [59–61]. Conservation measures aiming at reforesting open areas with native trees can speed up the process of forest restoration and regeneration [62, 63], and thereby mitigate the gap between the altitudinal shifts of El Oro parakeets and their food plants.

To enable dispersal and gene flow for El Oro parakeets in the future and, with it, to prevent a possible population decline due to habitat loss, it is crucial to not only aim at restoring or facilitating connectivity in a horizontal direction between forests in the same altitudinal band. Additionally, the vertical connectivity between forests in different altitudes has to be taken into account in order to allow for movement of parakeets into higher zones. In view of the high biodiversity in the Tumbes-Chocó-Magdalena hotspot and the similar altitudinal change of the endemic Ecuadorian tapaculo, it seems likely that many other cloud forest species are affected by climate change and the related range shift. Considering the high number of over 350 co-occurring bird species, of which 15 are threatened and 34 locally endemic [35], the El Oro parakeet has an important function as an umbrella species not only for the assessment of the upslope shift, but also for the targeting of sound conservation actions. We hypothesize that many other cloud forest species, including less mobile species like understory birds and amphibians, will greatly benefit from conservation measures aimed at restoring horizontal and vertical connectivity for the El Oro parakeet.

Our study yields general information about the potential risks that montane species face driven by climate change. In mountain ecosystems worldwide, species are undergoing range shifts caused by climate change [e.g., 15, 16, 64, 65]. Accompanying the upslope shift, a loss in range size can be aggravated by the disproportionally large decrease in suitable habitat and the arising barriers to connectivity. As a result, an upslope shift may cause drastic reductions in population size and genetic diversity, putting the long-term survival of montane species at risk. Our study provides a framework to assess the long-term persistence or extinction risk of mountain species in the face of increasing pressure from climate change.

## Supporting information

S1 Table List of models used in the IPCC WG1 AR5 Annex I: Atlas of global and regional climate projections (Stocker et al., 2013). (DOCX)

S1 Fig Relationship between temperature anomalies and extrapolated mean altitude of El Oro parakeets for the years 1985, 1986, 2002, 2011, and 2012. A temperature increase of 1°C resulted in an increase in altitude of 416 m (depicted by the blue line).
(TIF)
