# Supplementary material for: Projected impacts of climate change on habitat availability for an endangered parakeet
Source: PLoS One. 2018 Jan 24;13(1):e0191773. doi: 10.1371/journal.pone.0191773 (PMC5783391; doi:10.1371/journal.pone.0191773)
Supplement: S1 Table — (DOCX) [file pone.0191773.s001.docx]

**S1 Table** List of models used in the IPCC WG1 AR5 Annex I: Atlas of Global and Regional Climate Projections (Stocker et al., 2013)

| **CMIP5 Model Name** | **RCP2.6** | **RCP4.5** | **RCP6.0** | **RCP8.5** |
| --- | --- | --- | --- | --- |
| ACCESS1-0 |  | X |  | X |
| ACCESS1-3 |  | X |  | X |
| bcc-csm1-1 | X | X | X | X |
| bcc-csm1-1-m | X | X | X |  |
| BNU-ESM | X | X |  | X |
| CanESM2 | X | X |  | X |
| CCSM4 | X | X | X | X |
| CESM1-BGC |  | X |  | X |
| CESM1-CAM5 | X | X | X | X |
| CMCC-CM |  | X |  | X |
| CMCC-CMS |  | X |  | X |
| CNRM-CM5 | X | X |  | X |
| CSIRO-Mk3-6-0 | X | X | X | X |
| EC-EARTH | X | X |  | X |
| FGOALS-g2 | X | X |  | X |
| FIO-ESM | X | X | X | X |
| GFDL-CM3 | X | X | X | X |
| GFDL-ESM2G | X | X | X | X |
| GFDL-ESM2M | X | X | X | X |
| GISS-E2-H p1 | X | X | X | X |
| GISS-E2-H p2 | X | X | X | X |
| GISS-E2-H p3 | X | X | X | X |
| GISS-E2-H-CC |  | X |  |  |
| GISS-E2-R p1 | X | X | X | X |
| GISS-E2-R p2 | X | X | X | X |
| GISS-E2-R p3 | X | X | X | X |
| GISS-E2-R-CC |  | X |  |  |
| HadGEM2-AO | X | X | X | X |
| HadGEM2-CC |  | X |  | X |
| HadGEM2-ES | X | X | X | X |
| immcm4 |  | X |  | X |
| IPSL-CM5A-LR | X | X | X | X |
| IPSL-CM5A-MR | X | X | X | X |
| IPSL-CM5B-LR |  | X |  | X |
| MIROC5 | X | X | X | X |
| MIROC-ESM | X | X | X | X |
| MIROC-ESM-CHEM | X | X | X | X |
| MPI-ESM-LR | X | X |  | X |
| MPI-ESM-MR | X | X |  | X |
| MPI-ESM-P |  |  |  |  |
| MRI-CGCM3 | X | X | X | X |
| NorESM1-M | X | X | X | X |
| NorESM1-ME | X | X | X | X |
|  |  |  |  |  |
| **Number of Models** | **32** | **42** | **25** | **39** |
