# Supplementary figures and images for: Projected impacts of climate change on habitat availability for an endangered parakeet
Source: PLoS One. 2018 Jan 24;13(1):e0191773. doi: 10.1371/journal.pone.0191773 (PMC5783391; doi:10.1371/journal.pone.0191773)

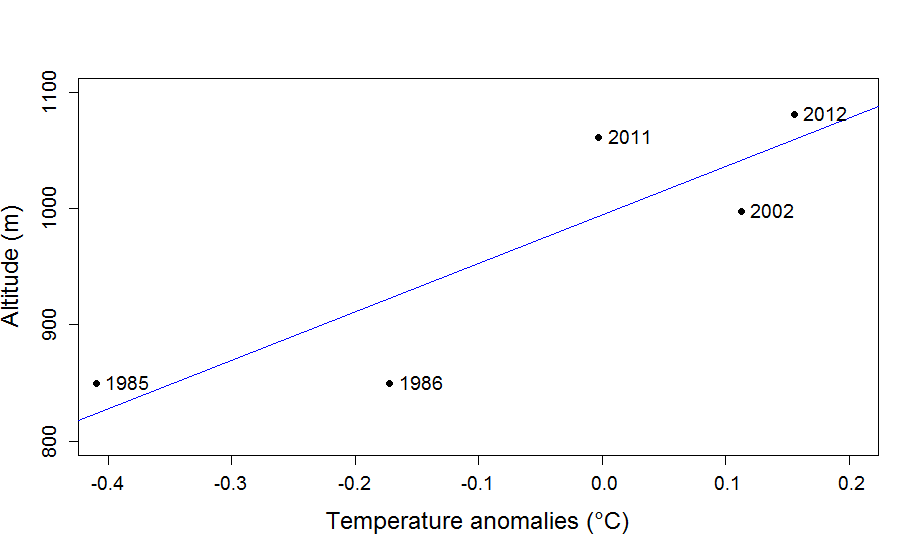

Supplement: S1 Fig — A temperature increase of 1°C resulted in an increase in altitude of 416 m (depicted by the blue line). (TIF) [file pone.0191773.s002.tif]
